# Evolution of Epileptiform Activity in Zebrafish by Statistical-Based Integration of Electrophysiology and 2-Photon Ca^2+^ Imaging

**DOI:** 10.3390/cells9030769

**Published:** 2020-03-21

**Authors:** Olga Cozzolino, Federico Sicca, Emanuele Paoli, Francesco Trovato, Filippo M. Santorelli, Gian Michele Ratto, Maria Marchese

**Affiliations:** 1 National Enterprise for Nanoscience and Nanotechnology (NEST), Istituto Nanoscienze Consiglio Nazionale delle Ricerche (CNR) and Scuola Normale Superiore Pisa, 56127 Pisa, Italy; olga.cozzolino@sns.it (O.C.); emanuelep.ep@gmail.com (E.P.); francesco.trovato@sns.it (F.T.); 2 Molecular Medicine, IRCCS Fondazione Stella Maris, Via dei Giacinti 2, 56028 Pisa, Italy; federico.sicca@fsm.unipi.it (F.S.); filippo3364@gmail.com (F.M.S.)

**Keywords:** epilepsy, zebrafish, statistical-based analysis, two-photon calcium imaging, electrophysiology

## Abstract

The study of sources and spatiotemporal evolution of ictal bursts is critical for the mechanistic understanding of epilepsy and for the validation of anti-epileptic drugs. Zebrafish is a powerful vertebrate model representing an excellent compromise between system complexity and experimental accessibility. We performed the quantitative evaluation of the spatial recruitment of neuronal populations during physiological and pathological activity by combining local field potential (LFP) recordings with simultaneous 2-photon Ca^2+^ imaging. We developed a method to extract and quantify electrophysiological transients coupled with Ca^2+^ events and we applied this tool to analyze two different epilepsy models and to assess the efficacy of the anti-epileptic drug valproate. Finally, by cross correlating the imaging data with the LFP, we demonstrated that the cerebellum is the main source of epileptiform transients. We have also shown that each transient was preceded by the activation of a sparse subset of neurons mostly located in the optic tectum.

## 1. Introduction

Epilepsy is one of the most common chronic neurological disorders and affects nearly 65 million people worldwide [1]. Since about one-third of patients show no significant improvement in current therapeutic treatments [2], there is a clear need for a better understanding of the disease and for more effective drugs. Zebrafish is an emerging model for the study of human neurological disorders, and for the identification of potential therapeutic targets [3,4]. Although the Central Nervous Systems (CNS) of mammalian and teleost display considerable neuroanatomical differences, several fundamental principles of brain development and function are evolutionarily well conserved [5], thus allowing to recapitulate in the fish many mechanisms and clinical features of brain disorders [6].

The molecular genetics of epilepsy cohorts is revealing an increasing number of genetic mutations that leads to the neurological disease. The functional study of the mutated genes can exploit the simplicity of the zebrafish model [7,8]. Both advanced genome editing [7,9] and the more rapid morpholino technique for the transient knockdown of specific genes [10,11,12] have been adopted to mimic human epileptic conditions in fish. Importantly, zebrafish models recapitulate many features of human pathologies, including behavioral aspects of the seizure phenotype, such as locomotor patterns and loss of posture [11,13]. Up to now, locomotion represents the main readout of the epileptic phenotype and the pattern and speed of swimming behavior of larvae can indeed be measured using automated locomotion-tracking [14] to provide information on seizure severity and on the outcome of administered drugs. Electrophysiological recordings [9,15,16,17,18] permit activity monitoring in intact larvae, but the analysis of the electrophysiology data relies mostly on their visual inspection. Moreover, it is often difficult to tell true epileptiform activity apart from physiological events, such as eye and tail movements [18] on account of the high sensibility of the electric signal to muscle activity due to the small dimension of the entire organism. Therefore, electrophysiology requires additional information for the unequivocal identification of the mutant phenotypes and for the evaluation of anti-epileptic compounds.

Recently, deep learning classifiers have been employed to identify electrophysiological events [19] and, although these methods are very fast and effective, they are unavoidably affected by the need of an a priori classification of epilepsy features and by the degree of completeness of the training dataset. A high-throughput local field potential (LFP) recording platform has also recently been used to analyze high-order statistical moments for an unsupervised detection of seizures [20]. However, these electrophysiological methods for seizure identification and classification are still prone to be affected by motion artefacts. Moreover, a complete physio-pathological interpretation of LFP recordings is hindered by the impossibility of identifying the sources of the electrophysiological signals and the spatial dynamics of epileptic activity of the underlying neuronal populations.

A complementary window on brain function is provided by imaging the fluorescence of genetically encoded Ca^2+^ indicators such as GCaMP6. Indeed, the zebrafish is an ideal model for the optical monitoring of brain activity given the small size of the larval brain and the availability of non-pigmented lines expressing GCaMP6. Ca^2+^ imaging provides information about neuronal activation at low temporal frequency, compared to LFP recordings, but with a very high spatial resolution [21]. Electrophysiological and behavioral assays on a GCaMP6 transgenic line have been combined with Ca^2+^ wide-field imaging to map activity at the level of wide brain regions, but the correlation of imaging data with LFP changes remained substantially qualitative [22]. Wide-field imaging of zebrafish neuronal activity allows us to describe distinct changes in local and long-range epileptic neuronal dynamics [23].

Here, we develop an unbiased and strictly quantitative method for the concurrent evaluation of LFP and 2-photon imaging data. We show how the statistics of the time series provided by the recordings of LFP and Ca^2+^ imaging can be used to classify activity in zebrafish either when seizures are induced by exposure to the pro-convulsant GABA_A_R antagonist pentylentetrazole (PTZ) [24], or by knocking down the *kcnj10a* gene, as in a model of the EAST (Epilepsy, Ataxia, Sensorineural deafness, and Tubulopathy) /SeSAME (Seizures, Sensorineural deafness, Ataxia, intellectual (Mental) disability, and Electrolyte imbalance) syndrome [11]. In the knockdown model, we also observed that pathological activity and neuronal recruitment revert under appropriate anti-epileptic treatment. Then, we demonstrate that the integration of Ca^2+^ imaging with LFP recordings pinpoints the specific regional sources of epileptiform activity. Finally, by complementing a ROI (Region Of Interest)-free approach with single neuron-based analysis, we determined the presence of two distinct cellular cohorts differentially contributing in the insurgence of the epileptiform discharges.

## 2. Materials and Methods

### 2.1. Zebrafish Lines and Maintenance

Adult male and female zebrafish Tg (neurod1:GCaMP6F) strain in the nacre (mitfa−/−) background were used for the study (kindly donated by Dr. Claire Wyart from Institut du Cerveau et de la Moelle Épinière, Paris, FR) [25]. Zebrafish embryos and larvae were maintained at 28 °C in embryo water on a 14/10 h light/dark cycle following standard procedures [26]. Zebrafish *kcnj10a* knockdown was generated as already described elsewhere [11,12]. To transiently knockdown the Kir4.1 function in zebrafish, we injected 0.5 ng of *kcnj10a* MO (Gene Tools, Philomath, OR, USA) targeting the splice site of the gene, into one- to two-cell stage embryos, as already reported [11].

### 2.2. Ethics Statement

All study protocols were approved by the Research Ethics Committee of the IRCCS (Istituto di Ricovero e Cura a Carattere Scientifico) Stella Maris Foundation, Pisa (Italy). Procedures involving *Danio rerio* were performed in accordance with the European Union (EU) Directive 2010/63/EU for animal experiments, and under the supervision of the Institutional Animal Care and Use Committee (IACUC) of the University of Pisa and the Italian National Research Council Institute of Clinical Physiology (CNR-IFC). Animals were always managed and injected according to the principles of Good Animal Practice as defined by the Italian animal welfare regulations. Every effort was made to minimize animal suffering and to use the minimum number of animals necessary to collect reliable scientific data.

### 2.3. Local Field Potential Recording and Pharmacological Treatments

Each 5 dpf (days post fertilization) larval zebrafish was placed in a 40 µL of egg water in the recording chamber with a transfer pipette, and then 200 µL of 1.2% low melting point agar were added in egg water. The chamber was transferred on the stage of a stereomicroscope for LFP recordings only, or on the stage of the two-photon microscope for the double recordings, as shown in Supplementary Figure S1. The local field potential (LFP) was recorded by a glass microelectrode (1–2 MΩ resistance) back loaded with extracellular recording solution and Sulforhodamine 101(Sigma-Aldrich, St. Louis, MO, USA) (0.1 mM diluted in 2 M NaCl) to allow imaging of the electrode tip under the two-photon microscope. Electrophysiological signals were amplified 1000-fold (EXT-02F, NPI Electronic), band pass filtered (0.1–1000 Hz) and oversampled at 5 kHz with 16-bit precision by a National Instruments (NI-usb6251) AD board controlled by custom made LabView software (National Instruments, Austin, TX, USA). Line frequency 50 Hz noise was removed by means of a linear noise eliminator (Humbug, Quest Scientific, North Vancouver, BC, CA). The electrode tip was placed under visual guidance in the medial tectal band of the midbrain at about 250 µm depth. At the end of the recording session, the position of the microelectrode was reconstructed together with the surrounding brain structures by means of 2-photon imaging (see Supplementary Video S1 for a three-dimensional (3D) reconstruction). Chemical treatments were performed by adding 100 µL of the PTZ (Pentylenetetrazol) or VPA (Valproic Acid) solution to the recording chamber near the larva. PTZ and VPA (Sigma-Aldrich St. Louis, MO, USA) were prepared in aquarium water at the concentrations of 30 mM and 2 mM, respectively.

### 2.4. Two-Photon Calcium Imaging

In vivo two-photon calcium imaging was performed on a Prairie Ultima Multiphoton microscope equipped with a mode-locked Ti:Sapphire laser (Coherent Chameleon Ultra II, Coherent, Santa Clara, CA, USA) and the power of excitation on the sample was <15 mW. Acquisitions were performed with spiral scanning at about 4 Hz with a water immersion lens (Olympus, 20×, 1.00 NA, Tokyo, JP) at a resolution of 512 × 512 pixels at an excitation of 920 nm. The imaging field was circular with a diameter of 607 µm. Imaging data were analyzed with ImageJ and custom Matlab code (Mathworks, Natick, MA, USA).

The motion of the fish is calculated from the time-lapse sequences as the difference between the position of the larva at each time point, computed as the area occupied by the fish and the median position of the larva itself, calculated over the entire time-lapse sequence.

### 2.5. Data Analysis

Data analysis of the LFP recordings was performed by custom Matlab code. The statistical analysis of the LFP power was performed as follows: (A) LFP recordings was filtered in the 30–95 Hz band. (B) The RMS (Root Mean Square) power was computed in a 250 ms sliding window that was translated on the trace 50 ms at a time to provide overlap between the samples. (C) The distribution of the logarithm of the RMS power was analyzed by an iterative procedure that computes the best fit normal distribution, removes the samples on the right tail (high power samples) from the original population and reiterates this procedure until mean and median of the residual population converge at a specific threshold. This distribution constitutes the Main Mode of the data. All the samples lying on the right tail constitute the Secondary Mode and the numerosity and the mean of this population are a measure of the extent of high-power electrophysiological events. The right tail of the distribution is formed by events farther than 2 standard deviations from the mean of the Gaussian distribution (see Supplementary Figure S2).

Imaging data were analyzed with custom Matlab code. The data were processed as follows: (A) The mean dark signal was subtracted from each time lapse stack and afterwards was binned 2 × 2 to decrease the size of the array. (B) The fluorescence fluctuation (ΔF/F0) was computed for each pixel by the equation:

ΔF(x,y,t)/F0 = (f(x,y,t) − 〈f(x,y)〉)/〈f(x,y)〉(1)

where f(x,y,t) is the fluorescence of each pixel (after dark subtraction and binning) and 〈f(x,y)〉 is the mean value. Our data were not affected by photobleaching and no correction was required. An alternative method uses a movable baseline window that computes the fluctuation with respect to a local baseline [27]. This method can correct from slow drifts of fluorescence but, in the presence of large Ca^2+^ transients, as happens during the movement (see Supplementary Figures S3 and S4), it causes a strong distortion of the response with the appearance of artifactual negative transients [28] and underestimation of the duration of the fluctuations. The ΔF(x,y,t)/F0 process has a mean value of 0 and it is normally distributed in the absence of significant Ca^2+^ events. In the presence of activity, a right tail emerges and the amount of Ca^2+^ fluctuations can be described in terms of the skewness of the distribution. The spatial domains of high Ca^2+^ activity were identified by the following procedure: (A) each sample from the ΔF(x,y,t)/F0 process was tested to ascertain the probability of belonging to the Gaussian distribution of the entire recording. If the sample falls within the baseline, distribution is set to zero, otherwise is set to 1 (Supplementary Figure S5). (B) The binary stack was low-pass filtered in both space and time. Positive pixels were retained if they lasted for a specified minimum time (3 frames throughout the study) and if they were surrounded by a definite minimum number of adjacent positive pixels (2 pixels). (C) The spatial domains were identified by clustering topologically connected pixels. This analysis was performed on the 3D stack x,y,t and not on the single frames by employing the ‘bwconncomp’ function included in the Matlab ‘Processing Image Toolbox’.

The neuron detection proceeds as follows: (A) The median projection of the entire stack was low-pass filtered (median filter ‘medfilt2’ 3 × 3) in order to reduce noise. (B) Convolutional cell-like filters (donut-shaped filters of size 10 and 7 pixels) were used to identify the coordinates of all neurons. (C) Around each set of coordinates, a circular ROI was drawn with a radius of 3 pixels (3.6 µm) to obtain the time series of the Ca^2+^ fluctuation for all identified neuron. (D) The time series ΔF/F0 was computed for each identified neuron, as described previously. The time series were prepared for the PCA (Principal Component Analysis) computation by normalizing them to unitary standard deviation. PCA was performed using the dedicated MatLab function on the entire imaging stack using each ROI as a variable and the time points as samples. The principal components that explain a high percentage of variance are determined by the coordinated activity of a neuronal population. Each neuron is associated by its own weight (“loading”) in terms of participation to the activity pattern described by each PC. We decided to limit this analysis on the first 10 PCs, since they are sufficient to explain 41.8% of the variance for the MO (Morpholino) and 64.5% for the PTZ. The principal component that best describes the dynamic in the high-frequency band of the LFP has the highest value of cross-correlation coefficient at lag zero (Supplementary Figure S9B, green), while the one PC representing the activity preceding the ictal-like events is the positive peak with the closest negative lag with respect to lag zero (Supplementary Figure S9B, magenta). We define these two descriptors as “epileptiform” (Ep) and “pre-epileptiform” (pre-Ep).Moreover, after this dimension, we reached a plateau where each additional PC can explain less than 1% of the variance (Supplementary Figure S9C). 

The mean cross-correlogram of the selected components (pre-Ep and Ep) with the RMS power were computed in a window of 25 s around the paroxysmal electrophysiological events.

We wrote custom code to create the maps that show the neuronal population involved in the epileptic and pre-epileptic responses. The pipeline was: (A) the logarithm of RMS in the 30–95 Hz band was interpolated at the same mean time point of each frame. (B) The first 10 principal components were cross-correlated with the interpolated RMS power. (C) From the cross-spectra, we selected the two spectra with the criterion explained above. (D) A map was computed associating the normalized loading of the two selected PCs of each time series with the xy position in the imaging recording. Neurons with a negative loading were removed.

To align data from different larvae, we performed a non-rigid registration to a reference brain mask with the algorithm by D.J. Kroon (https://it.mathworks.com/matlabcentral/fileexchange/20057-b-spline-grid-image-and-point-based-registration?s_tid=prof_contriblnk). The reference brain mask was drawn manually in ImageJ.

The collective maps of Figure 6D are computed as follows: first, we computed the normalized loading of each neuron as:

Normalized loading _(i_neuron)_ = (loading _(i_neuron)_) / M_load_ (2)

where M_load_ represents the maximum loading value (on all neurons) for that PC. The neurons that participate during both pre-Ep and Ep periods were removed in order to focus on neurons belonging exclusively to only one functional class. Next, we computed the activation maps for each epileptiform event, and we averaged all maps for each fish. Finally, after non-rigid registration, we computed the maximum projection of the 4 maps representing 4 animals for each model.

### 2.6. Statistical Analysis

Plots and statistics were obtained by using Prism (GraphPad) or Origin 8. All data were represented with the appropriate box plots and were analyzed with the non-parametric Mann–Whitney test. Significance levels were indicated as * = *p* ≤ 0.05; ** = *p* ≤ 0.01; *** = *p* ≤ 0.001; **** = *p* ≤ 0.0001.

## 3. Results

### 3.1. Statistical Analysis of LFPs

Zebrafish larvae (120 h post-fertilization) were restrained in low melting point agar and the electrode was placed along the medial tectal band, as indicated in Supplementary Figure S1A,B. Imaging was performed at a depth of 140–160 µm under the surface, thus providing an optical section that includes neuronal populations belonging to optic tectum, cerebellum and hindbrain (Supplementary Figure S1C, see Supplementary Video S1 for a 3D reconstruction). We analyzed three experimental groups: wild-type (WT) larvae, WT larvae treated with PTZ as a model of pharmacologically induced seizures and the morphants of *kcnj10a* as a model of EAST syndrome [11], a brain disease characterized by spontaneous seizures. For each group, after attaining a stable electrophysiological signal, the LFP was recorded for at least 30 min.

Supplementary Figure S2A,B show the full band and the band pass (30–95 Hz) LFP-recorded in a morphant zebrafish. The LFP was analyzed by computing the statistics of the root mean square power of the signal in the same bandwidth. The power of the band passed data was sampled in windows 250 ms long that were shifted 50 ms until covering the entire data set (Supplementary Figure S2C). Supplementary Figure S2D shows the distribution of the logarithm of the power of the resting state activity (magenta interval) that it is well represented by a normal distribution. When we included large spontaneous events, we observed a bimodal distribution, with most power values in the Gaussian-like mode (dubbed Main Mode, MM) and a tail containing high-power events (Secondary Mode, SM) that are rare but of high physio-pathological relevance (Supplementary Figure S2D). As a metric of the statistics of electrophysiological activity of each experimental group, we used the mean of the power of the MM and the difference between the means of the Secondary and Main modes (ΔSM-MM, see gray bar in the right panel of Supplementary Figure S2D).

**Figure 1:**
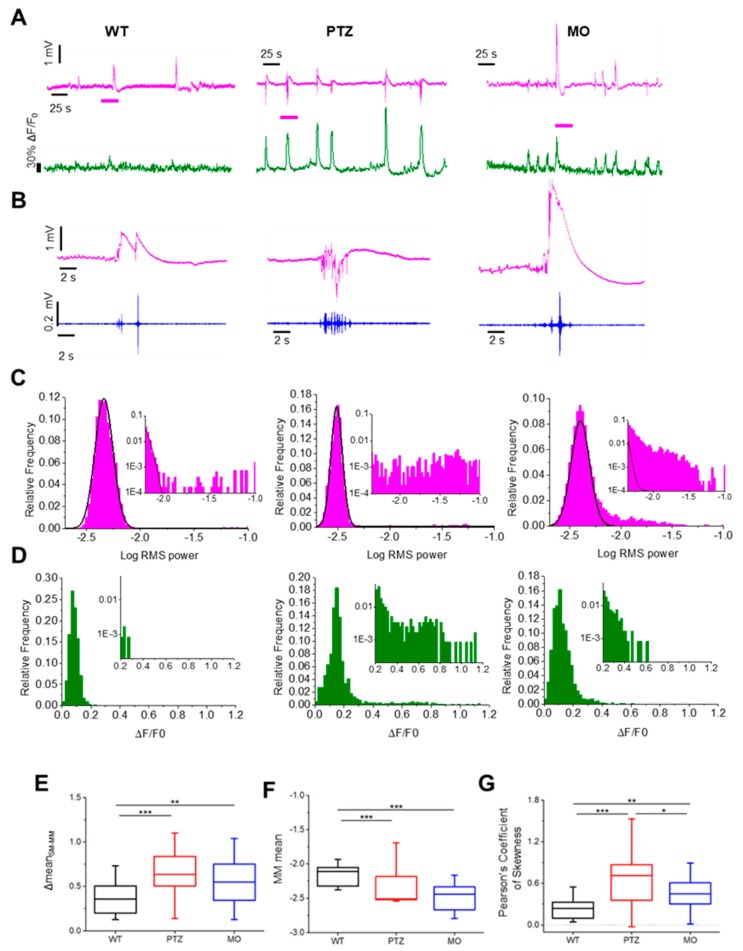
Statistics of the local field potential (LFP) spectral power and Ca^2+^ recordings in the three experimental groups. (**A**) Comparison of the statistics of LFP and whole brain Ca^2+^ fluctuations recorded in the three experimental groups, wild-type (WT) as control zebrafish, PTZ (Pentylenetetrazol) after treatment with pentylenetetrazole and MO (*kcnj10a* splice-morpholino) morphants of *kcnj10a*. The LFP transients in the PTZ and MO groups are associated to generalized positive fluctuation of the Ca^2+^. (**B**) Magnification of the LFP events is indicated by the magenta bars in panel A. The magenta traces show the full band data, while the blue traces show the data band passed in the 30–95 Hz range. (**C**) Statistical analysis of the electrophysiological activity. (**D**) Gaussian-like distribution of the ΔF/F0 values in the three models. (**E**) Difference between the Secondary Mode (SM) mean and the mean of the Main Mode (MM) (ΔmeanSM - MM). (**F**) Comparison of the MM mean of the three different models. (**G**) Statistics of the Pearson’s coefficient of skewness of the distribution of the fluorescence fluctuations, ΔF/F0, integrated on the entire optical section. Statistics have been cumulated from 5 recordings for each larva for each condition (* *p* ≤ 0.05,** *p* ≤ 0.01, *** *p* ≤ 0.001).

Figure 1A,B shows representative recordings from WT zebrafish in the control condition, after treatment with PTZ and from a *kcnj10a* morphant (MO). The statistical analysis of the power distribution (Figure 1C,E) shows that the ΔSM-MM values are far larger in the PTZ and MO groups, meaning that these pathological conditions are characterized by a higher frequency of high-power events. The statistics of the resting state is defined by the power of the Main Mode, as represented in Figure 1F: larvae treated with PTZ showed a larger heterogeneity compared to controls, whereas morphants showed a smaller power, indicating a lower spontaneous activity in between large events. The statistics of the entire data population (n = 5 fish for each group) reveals that the PTZ and MO groups manifest frequent large events characterized by long high-frequency discharges consistent with epileptiform activity. Conversely, the large and sustained low-frequency transients present in WT larvae (Figure 1B) are not accompanied by high-frequency oscillations, demonstrating a substantial difference in the underlying mechanism. This second type of events complicates the interpretation of the LFP signal affecting the automated seizure detection process [19]. A possible explanation of a scarce occurrence of high-frequency oscillations in control larvae is that large transients at low frequency are caused by tail flicks with the recruitment of a limited neuronal cohort that does not drive enough synchronous activity to be detected by the LFP.

### 3.2. Combined LFP Recording and Calcium Imaging

Next, we combined the LFP recordings and Ca^2+^ imaging performed in transparent larvae expressing the Ca^2+^ sensor GCaAMP6f [25] to estimate the degree of neuronal recruitment associated with the LFP transients. Figure 1A shows the representative LFP recordings aligned to the corresponding Ca^2+^ fluctuation recorded on the whole brain. Data from PTZ- and MO-treated zebrafish show large Ca^2+^ transients tightly correlated with the LFP. In contrast, the WT does not show such a clear correlation, suggesting that the low-frequency LFP transients are associated to the recruitment of a very limited network (see also Supplementary Video S2–S4).

The distribution of the fluorescence fluctuations (ΔF/F0) (see the Material and Methods Section) of the entire optical section must reflect the extent of neuronal recruitment for each experimental group. As seen in the statistical analysis of the LFP power, high-activity transients skew the distribution toward the right (Figure 1D). This effect can be quantified by the Pearson’s coefficient of skewness, defined as:

∂ = (3(M − m)) ⁄ σ(3)

where M is the mean, m is the median and σ is the standard deviation of the distribution. The PTZ treatment and the gene knockdown of *kcnj10a* cause a substantial increase of the skewness, indicating the recurring presence of large fluctuations of intracellular Ca^2+^ (Figure 1G), nicely reflecting the observations made on the LFP statistics.

### 3.3. Spatio-Temporal Evolution of Calcium Activity

Traditional approaches to the analysis of Ca^2+^ imaging [22,28] proceeds by designating regions of interest (ROIs) to compute the temporal evolution of the Ca^2+^ signal. This analysis is suitable for a single cell-based approach, but it falls short when applied to larger brain areas, since the selection of interesting areas is heavily affected by subjective judgment of the operator. Initially, we have performed a purely statistical analysis of the fluorescence fluctuations. Supplementary Figure S3A shows that the fluctuation distribution of each pixel is skewed (see, for example, the orange frame in Supplementary Figure S3, panel A), with a right tail associated to positive Ca^2+^ transients. The more active a pixel is, the larger the right tail and the overall distribution differs more from the normal distribution that is representative of pixels containing noise only (green frame in Supplementary Figure S3, panel A). The time series can be summarized by a map, where each pixel is color-coded depending on the degree of asymmetry of the distribution of the fluctuations of fluorescence (Supplementary Figure S3B). These maps allow the rapid and unbiased analysis of an entire imaging session, providing important clues for the identification of the brain regions undergoing Ca^2+^ oscillations, independently of the intensity of the basal fluorescence. Supplementary Figure S3B shows that the brain regions with the highest Ca^2+^ fluctuation are much larger in the PTZ-treated larvae and in morphants. In contrast, WT larvae show the recruitment of very small regions (white arrow in Supplementary Figure S3B). A detailed analysis of WT data reveals that these spatially segregated events are associated with low-frequency transients of the LFP and with tail flicks, as witnessed by the movement of the head profile (Supplementary Figure S4 and Supplementary Video S5).

These maps do not provide any information about the spatio-temporal evolution of activity, but we can exploit the temporal statistics of each pixel to obtain a dynamic representation of the high Ca^2+^ domains. The following analysis allows us to classify each pixel depending on the likelihood that the instantaneous value of the fluorescence fluctuation (ΔF/F0) belongs to the right tail of the distribution, as determined by a statistical-based threshold criteria (Supplementary Figure S5). Thus, each frame of the imaging sequence is transformed by setting all pixels that satisfy the threshold condition to 1 and all pixels that fall within the normal baseline distribution to 0 (see the Materials and Methods Section for details and Supplementary Figure S5).

This analysis provides a dynamic representation of the recruitment of brain territories during activity transients (Supplementary Figure S6). This way of representing Ca^2+^ data enlightens a drastically different spatial structure of the events recorded in the WT, PTZ and MO groups. The WT shows a very limited recruitment associated to tail flicks (see also Supplementary Figure S4), as quantified by the small number of pixels falling over the threshold. In contrast, the PTZ treatment causes the recruitment of very large territories as the seizure propagates to the entire brain. The morphants shows an intermediate situation in which recruitment is limited to patchy regions distributed on most of the midbrain and part of the hindbrain.

The reconstruction of the three-dimensional (3D) clusters is performed by clumping together active voxels with a criterion of topological nearness extended to the x, y, t space (see the Material and Methods Section). Supplementary Figure S7 shows the passage from the binarized images to the identification of the 3D clusters. This procedure allows us to visualize the active regions in space-time and to produce a metric describing the properties of the clusters (position, duration, volume, surface).

**Figure 2:**
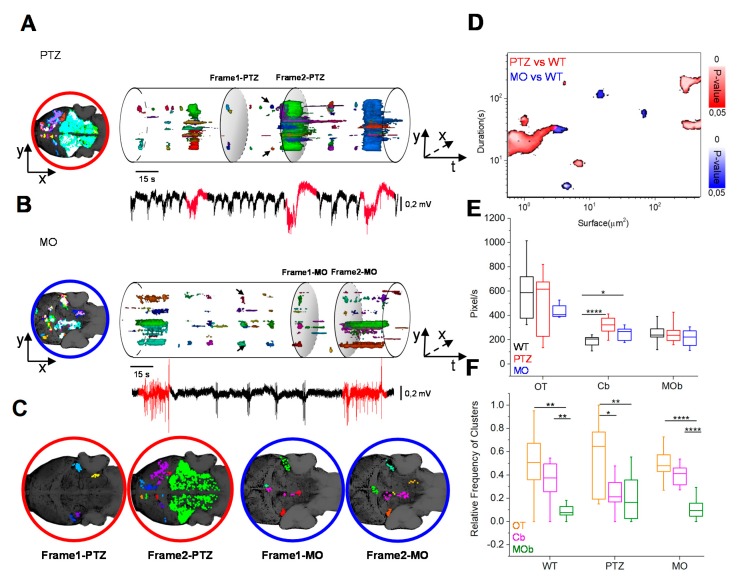
Dynamic evolution of the Ca^2+^ domains. (**A,B**) The volume representation depicts the active pixels from the binary stacks, clustered in domains of different colors of PTZ and MO larvae. The frame on the left is the projection of active domains during the entire period. The LFP events in red have high Ca^2+^ recruitment (>700 voxels). (**C**) The shaded sections in A and B are the spatial patterns of activation during the interictal-like events (frame 1) and the generalized events (frame 2) in the PTZ (red) and MO (blue). (**D**) Difference of the spatio-temporal morphology of PTZ and MO clusters with respect to WT clusters. The maximum surface occupied by Ca^2+^ clusters is plotted in relation to their duration. The color bars represent the *p*-value of the difference with the WT model. We report only the significative differences (*p*-value ≤ 0.05). 4 recordings for each larva, n = 3 larvae for each condition. (**E**) Comparison of the amounts of detected voxels for the three models in different anatomical regions. (**F**) Anatomical localization of the origin of the recruited neuronal clusters with volume > 300 voxels. The data have been normalized to the total number of active domains measured in each recording session (* *p* ≤ 0.05, ** *p* ≤ 0.01, *** *p* ≤ 0.001, **** *p* ≤ 0.0001).

Figure 2A,B show the three-dimensional Ca^2+^ domains recorded in two imaging sequences obtained from PTZ and MO larvae (see the 3D reconstructions in Supplementary Videos S6–S8). We measured the volume of the active regions recorded in all larvae and Figure 2D quantifies the spatio-temporal size of active clusters of PTZ and MO, with respect to the WT. The PTZ-treated larvae present mainly short, spatially-confined events, and longer, widely spread events, while in the MO larvae, the electrophysiological transients are associated to the activation of domains of different duration that never reaches the dimensions of the large PTZ seizures (Figure 2C).

In Figure 2E, we compared the degree of activity in the three main anatomical districts showing that the main difference in the activation is in the cerebellum, as also indicated in the maps of Supplementary Figure S3.

The long seizures covering a large fraction of the brain (see Figure 2A,C, Frame 2-PTZ) exhibited by the PTZ model show a pathophysiological coherence with the reduced efficacy of the GABAergic (gamma-Aminobutyric acid) system. Interestingly, these generalized events were never observed in the MO larvae.

Outside of the sporadic, generalized events, activity during PTZ treatment is characterized by quasi-periodic transients that recruit only small domains, with characteristic volumes in the range of 300–700 voxels, localized in the lateral areas of the cerebellum (see arrows in Figure 2A and Frame 1-PTZ in panel C). The MO larvae also show quasi-periodic activation of territorially limited domains mostly located nearby the brain medial tectal band and in the cerebellum (see arrows in Figure 2B and Frame 1-MO in panel C). The quasi-periodic nature of these territorial limited events suggests them as interictal-like discharges. Finally, we extracted all the transients larger than 300 voxels and we classified the spatial origin of each event according to the division in macro areas outlined in Supplementary Figure S1C. Figure 2F shows that the optic tectum has the higher number of spots, giving rise to activity clusters.

### 3.4. Valproate Treatment Attenuates Seizures in Morphant kcnj10a

**Figure 3:**
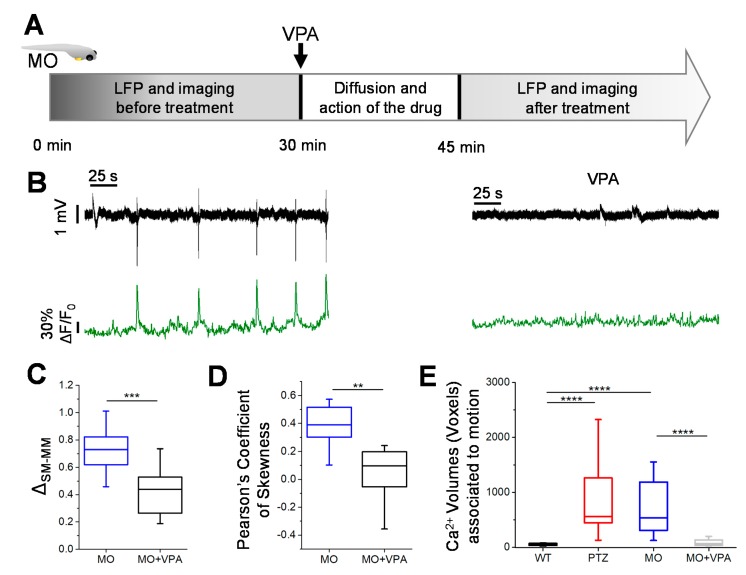
Valproate treatment. (**A**) Valproate (2 mM) was administered to MO *kcnj10a* larvae after 30 min of baseline recording of LFP and two-photon imaging. After a diffusion time of 15 min, the double recording was resumed. (**B**) Alignment of LFP and ΔF/F0 calcium traces in MO VPA( Valproic Acid)-treated larvae. (**C**) The analysis of the statistical distribution of the electrophysiological signal showed a clear effect of VPA in decreasing the energy of the LFP events. (**D**) The skewness of statistical distributions of Ca^2+^ fluctuations decreased during VPA treatment. (**E**) During VPA treatment, movements of the larvae did not correlate with a generalized increase of calcium signal. Therefore, VPA treatment led to a physiological phenotype, where tail flicks are associated not to a convulsive seizure but to the activation of a limited neuronal circuitry. 2 recordings for each larva, n = 3 larvae for each condition (* *p* ≤ 0.05, ** *p* ≤ 0.01, *** *p* ≤ 0.001, **** *p* ≤ 0.0001).

To validate our approach in the context of drug screening, we evaluated the effect of the neuroactive molecule valproic acid (VPA) on the resting state activity of the morphant larvae. In these experiments, we performed simultaneous LFP recording and 2-photon imaging for a 30 min baseline before administration of VPA. After a diffusion time of 15 min, we resumed the double recording (Figure 3A,B).

The statistical analysis of the LFP spectral power showed that VPA drastically reduced the secondary mode power, indicating a subsidence of interictal-like activity (Figure 3C). This effect was also confirmed by the analysis of the Ca^2+^ fluctuations that were drastically reduced during the VPA treatment (Figure 3D). Previously, we observed that in normal larvae, spontaneous movements were associated to small high-frequency transients and to the recruitment of very small Ca^2+^ domains (Supplementary Figure S4). In contrast, in the epilepsy models, movement is always associated to large Ca^2+^ domains, suggesting convulsive events (Figure 3E). After treatment with VPA, the large Ca^2+^ events ceased, and the size of active domains associated to the larva motion recovered a physiological value similar to the controls (Figure 3E).

### 3.5. Localization of the Sources of the LFP Transients

**Figure 4:**
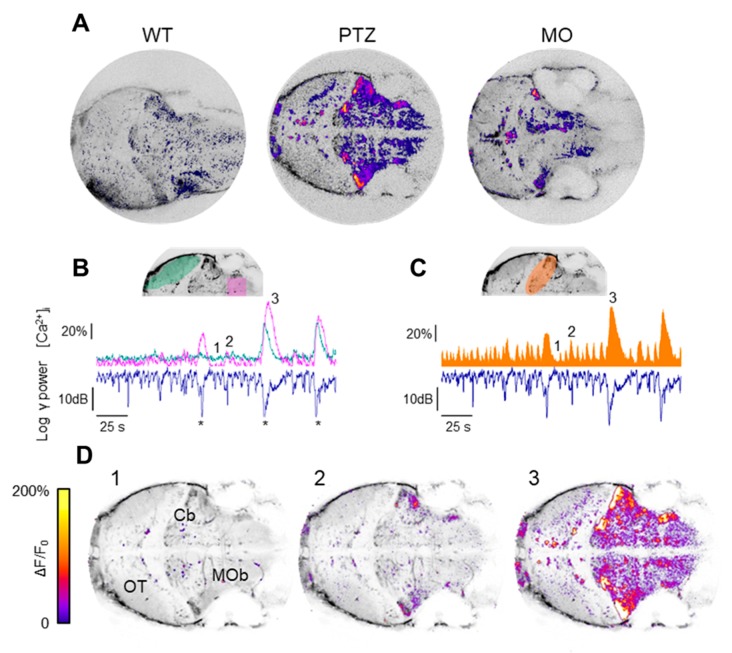
Localization of the sources of the LFP transients. (**A**) Maps obtained by computing the cross-correlation of the Ca^2+^ fluctuations of each pixel with the logarithm of the RMS (Root Mean Sqaure) power in the band 30–95 Hz in the three experimental models. (**B**) Example of recordings from a PTZ-treated larva where the correlation between the largest electrophysiological events (labelled by an asterisk) and Ca^2+^ activity measured in the OT (green trace) is apparent, and in the MOb (pink trace). (**C**) Ca^2+^ fluctuations measured in the Cb (orange trace, represented as an area to better appreciate details) correlate with both the large RMS events and the smaller interictal-like transients. (**D**) ΔF/F0 of the frames corresponding to baseline electrophysiological activity (labelled 1 in the above panels), to a small interictal event (2), in which Ca^2+^ activity is sparse and mostly localized in the Cb, and to a large electrophysiological event (3) associated to generalized Ca^2+^ activation. The overlay shows ΔF/F0 fluctuations larger than 10% over-imposed on the mean projection of the image stack.

The identification of the local circuits generating specific features of the LFP is a debated issue, also due to the lack of a general method to ascribe transient episodes of high-frequency activity to specific brain regions. Given the small size of the zebrafish brain, we reasoned that it should be possible to identify the putative sources of the LFP transients by computing the cross-correlation of the Ca^2+^ fluctuations with the LFP. The cross-correlation between the Ca^2+^ fluctuations of each pixel with the logarithm of the RMS power is expressed by the following equation:

(4) $$C\left(x,y\right)=\overset{L}{\underset{l=-L}{\sum }}\overset{T}{\underset{t=0}{\sum }}\log\left(RMS\left(t-l\right)\right){\frac{\Delta F\left(t\right)}{F_{0}}}_{\left(x,y\right)}$$

where *C*(*x*,*y*) is the cross-correlation map, *L* indicates the half width of the central peak of the cross-correlation spectra and *T* is the duration of the recording (Supplementary Figure S8). Figure 4A shows that the cross-correlation varied widely across the imaging field, being higher in the regions that contributed more to the LFP signal. The cerebellum is very prominent in both the PTZ and MO larvae.

The significance of the cross-correlation maps is clarified by comparing the Ca^2+^ fluctuations measured in different brain regions of the PTZ larva, with the RMS power of the LFP (Figure 4B,C). The fluctuations observed in regions with moderate cross-correlation are rather uncoupled from the LFP, except during the large generalized events (Figure 4B and compare with the correlation map of the PTZ-treated larva in panel A). In contrast, the fluctuations measured in the entire cerebellum show a very high degree of correlation not only during the large generalized events, but also during the small interictal-like events (Figure 4C,D).

**Figure 5:**
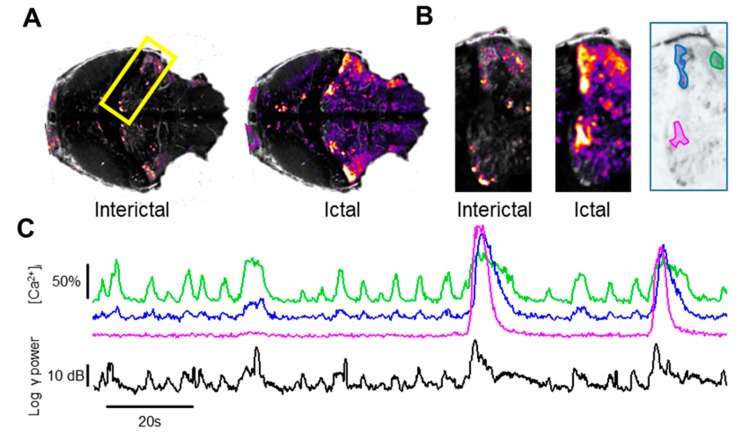
Identification of the micro-circuitry associated with different epileptic-like events. (**A**) Cross-correlation maps computed during two different intervals in th presence of only interictal activity (left) or during large ictal events (right). The cross-correlation maps have been normalized to the respective maximum value. (**B**) Details of the Cb, as outlined by the yellow rectangle (300 μm high) in panel A, showing the different sources of LFP signal during these two different classes of events. The finder map on the right shows three areas belonging to the cerebellum that are differentially active during interictal and ictal-like events. (**C**) ΔF/F0 measured in the three areas outlined by the corresponding colors in B. While all areas respond to the three large ictal events, the neuronal territory involved in interictal activity (green area and trace) is far more limited.

The cross-correlation analysis confirms that the small Ca^2+^ transients are not random fluctuations but reflects the activation of a local circuitry confined within the cerebellum that leads to the transient episodes of hypersynchronous activity observed in the LFP. The cross-correlation maps computed during periods of interictal activity are characterized by the sparse activation of a smaller territory compared to the more diffused recruitment observed during the large ictal events (Figure 5A). Moreover, we identified a small region that is differentially activated during the interictal bursts (Figure 5B), suggesting that distinct local circuits are active in these different epileptiform episodes, as shown in Figure 5C.

### 3.6. The Timing to Epileptiform Bursts Identifies Two Classes of Neurons

To study the dynamic at single cell resolution, we segmented the images by convolving the image stacks with a circular kernel (see the Materials and Methods Section). The detected regions correspond to the cell bodies of isolated neurons, and we computed the fluorescence fluctuations associated to each cell. The comparison of the Ca^2+^ dynamic of all neurons in an image stack with the RMS power of the LFP in the 30–95 Hz band (Supplementary Figure S9A) demonstrated the presence of two different activity patterns that are stereotyped during most transients. A large set of neurons show substantial activity during each LFP epileptiform transient (Supplementary Figure S9A, green arrowhead) whereas a smaller population is active just before of the LFP burst (magenta arrowhead).

**Figure 6:**
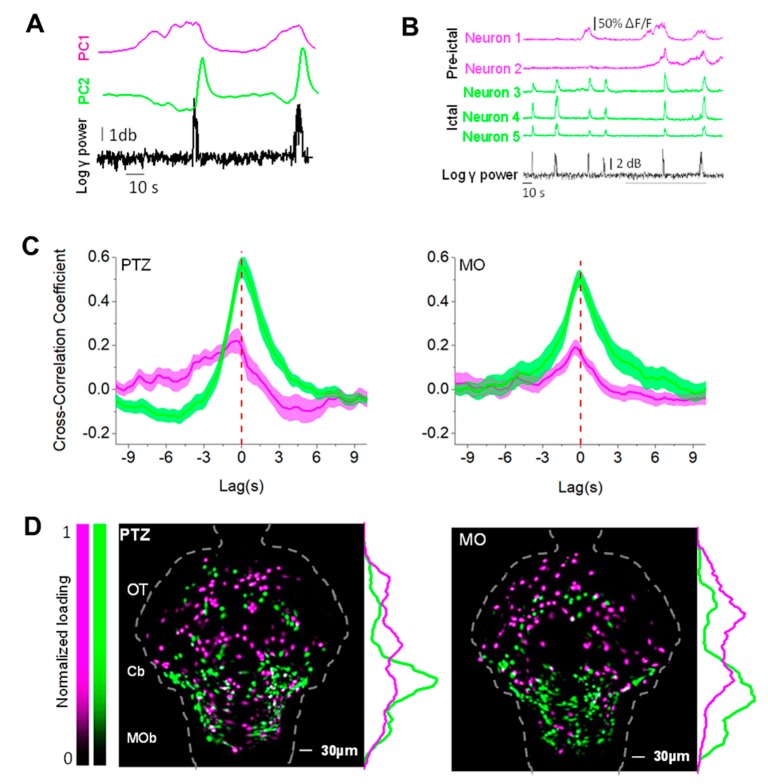
Principal component analysis (PCA) analysis identifies two different neuronal populations. (**A**) Population activity profiles represented by the first two PCA components describing the Ca^2+^ dynamics of single neurons. The black trace represents the corresponding RMS power. (**B**) Representative ΔF/F0 activity selected from single neurons best described by either PC1 (magenta) or by PC2 (green) in a PTZ-treated larva. PC1 neurons’ activation precedes the LFP bursts, whereas PC2 neurons activate during the LFP peaks. The grey bar indicates the events magnified in panel A. (**C**) Mean cross-correlograms of the two selected PCs with the aligned RMS power (n = 15 events from 4 morphants and n = 16 events from 4 PTZ larvae). (**D**) Cumulative maps for the PTZ (n = 4) and the MO (n = 4) larvae. Each cell is represented by its normalized loading (see the Materials and Methods Section) with respect to the PC1 and PC2. The plots on the right represent the normalized signal (arbitrary units) of the two-cell population along the rostro-caudal axis. The distribution of the two functional populations are significantly different in both models (*p* = 0.002 for the morphant and *p* < 0.0001 for the PTZ, two-samples Kolmogorov–Smirnoff test). In order to standardize the images, we registered the maps into a reference brain mask using a non-rigid image registration algorithm (see the Materials and Methods Section).

This qualitative analysis supports the idea that there are two neuronal populations that activate sequentially around the epileptiform bursts. We performed the principal component analysis (PCA) of the Ca^2+^ fluctuations of all the identified neurons in order to extract different shared activity profiles in the dataset. Then, we identified the principal components (PCs) that best represent these two behaviors. One PC represents the neurons active during the epileptiform (Ep) transients and the second represents the neurons active before the transient (preEp) (Figure 6A–B and Supplementary Figure S9D) (see the Materials and Methods Section for details). The two-mean cross-correlograms (Figure 6C) between the RMS power of the electrophysiological trace and the pre-Ep (magenta traces) or the Ep (green traces) components show that the dynamic of the pre-Ep neurons is different with more pronounced anticipation of the LFP event in the PTZ compared to the MO.

In order to find the topological organization of the neurons that participate to each of the two states (Figure 6B), we computed maps (see the Materials and Methods Section) showing the pre-Ep set of neurons in magenta and the Ep neurons in green.

Figure 6D shows only the neurons that fall in one of the two populations. These collective maps of all the PTZ and MO larvae are remarkably similar concerning the Ep population, localized in the cerebellum (Cb) and hindbrain (medulla oblongata, MOb). The main difference between the two models consists in spatial distribution of the pre-Ep group. In the morphants, it is more clustered in the OT, while this component does not show any clear spatial patterning in the PTZ (Pentylenetetrazol).

## 4. Discussion

The treatment of epilepsy is still challenging, since only 70%–75% of the patients achieve a satisfactory seizure control under antiepileptic drugs [29]. A quest for more effective therapies can rely on the simplicity of the zebrafish model provided that recordings are analyzed with a sufficient sensitivity to attain a robust assessment and classification of all epileptic manifestation. In this study, we offered new insights to identify the sources of the LFP changes and to dissect the spatio-temporal dynamics of epileptiform activity in PTZ-treated and *kcnj10a* morphant larvae. We employed the joint analysis of LFP and Ca^2+^ imaging recordings based on the statistics of the dataset, independent on a priori hypothesis on what is the signature of epileptiform activity and on where are the active regions.

Our method is capable to capture small, hypersynchronous events, and this high sensitivity is also crucial for the quantitative characterization of genetic models of epilepsy where seizures are very rare or absent, including models involving epilepsy-related cognitive decline. Indeed, inter-ictal like activity may play a pathological role [30] in several human syndromes, disrupting brain activity and computation, thus leading to cognitive impairment [31]. Our recordings from the MO group show that most epileptiform events have a smaller Ca^2+^ fingerprint compared to PTZ, since activity is confined in limited domains that do not extend to the entire optic section (see Figure 2C) and have limited duration. This inter-ictal like behavior of the EAST syndrome model is consistent with some of the pathomechanisms underlying intellectual disability [32] and with another clinical feature of EAST syndrome, ataxia, which is also recapitulated in the MO model that shows an almost complete lack of motor activity outside of the inter-ictal burst (Figure 3, [11]). Both the physiological movements and brain activity in MO are rescued by VPA treatment (Figure 3).

Our results point to the cerebellum as the main anatomical region involved in the generation of epileptiform signal (Figure 4 and Figure 5). The degree of the involvement of the cerebellum in epileptic mechanism is still under debate [33], even if recent optogenetic-based experiments demonstrate the possibility to alter the frequency and the direction of cortical seizures acting on the cerebellum [34]. In this study, Krook-Magnuson et al. [35] also demonstrates the existence of different circuits responsible for seizure generation and seizure maintenance. Their findings are consistent with our PCA analysis, since we identified a sparse population of neurons, mostly located anteriorly to the cerebellum, that activates before the epileptiform discharges. The time scale of these pre-seizure activities we found is in agreement with the temporal window that allows the prediction of epileptic seizures based on consideration of EEG (Electroencephalography) statistical properties through extreme events theory [35].

The sensibility necessary to unravel the finer features of the aforementioned epileptic models relies on the complementary information of the electrophysiology and imaging. The LFP has very high temporal resolution and, given the small size of the zebrafish brain, reports an integrated signal of brain activity but no indication on the localization of the sources. Ca^2+^ imaging is intrinsically much slower but carries a spatially resolved indication about the territories active at any given time. Studies that rely on only one modality are intrinsically limited and cannot capture the full complexity of epileptic activity. Recent developments include multi-electrodes platforms for the parallel LFP recording from multiple larvae [15,17,18,20]. These methods are extremely useful for the screening of antiepileptic drugs, but the presence of a large LFP transient is not sufficient to define an epileptic event. For example, the electrophysiological activity associated to motion (see Figure 1B, left panel) could be misinterpreted as a seizure, whereas Ca^2+^ imaging demonstrates how this kind of activity systematically corresponds to a spatially limited recruitment of a sparse neuronal cohort, a reflection of normal physiological activity (see Supplementary Figure S4). On the other hand, Turrini et al. employed Ca^2+^ imaging in the entire zebrafish brain by means of wide-field microscopy without a quantitative evaluation of the parallel recordings of electrical activity. This study provided some interesting readouts but did not identify the sources of the LFP transients [22].

Liu and Baraban exploited the complementarity of these techniques by an operator-assisted strategy for the identification of meaningful electrophysiological events followed by manual segmentation of the regions of interest for the analysis of the Ca^2+^ fluctuations [28]. Possibly for this reason, in the PTZ-treated larvae, this study only reported the generalized ictal events that involve a large fraction of the brain, while failing to identify the subtle, yet physio-pathologically relevant, interictal-like activity that spans a large fraction of the recorded period (see Figure 2, Figure 4 and Figure 5). Our approach can be easily extended to other experimental paradigms and models. For example, wide-field recordings of Ca^2+^ activity in zebrafish or mice [36] can be analyzed for the statistical extraction of the significant functional domains in a completely unbiased way. Similarly, the vast dataset obtained by light sheet imaging [37] could be classified and clustered based on the statistical properties of each voxel time series.

## Supplementary Materials

The following are available online at https://www.mdpi.com/2073-4409/9/3/769/s1: Figure S1: Experimental model, Figure S2: Statistical analysis of the LFP signal, Figure S3: Computation of the maps of Ca^2+^ activity, Figure S4: Activity and motion, Figure S5: Computation of the binarized imaging data, Figure S6: Spatio-temporal evolution of the recruitment of high Ca^2+^ domains in the three experimental groups, Figure S7: Volume identification, Figure S8: Computation of the cross-correlation maps, Figure S9: PCA analysis. Video S1: 3D Reconstruction of a zebrafish brain. A Z-stack was acquired from the brain surface down to a depth of 160 µm collecting one optic section every 5 micron, thus providing an optical section that includes neuronal populations belonging to optic tectum, cerebellum and hindbrain. The micro-electrode used for LFP recordings has been filled with rhodamine and it is visible in red. Video S2: Combined LFP and Ca^2+^ imaging recordings performed in a WT larva showing the degree of neuronal recruitment associated with the LFP transients. Video S3: Combined LFP and Ca^2+^ imaging recordings performed in a larva after PTZ treatment showing the degree of neuronal recruitment associated with the LFP transients. Video S4: Combined LFP and Ca^2+^ imaging recordings performed in kncj10a morphant larva showing the degree of neuronal recruitment associated with the LFP transients. Video S5: Ca^2+^ imaging during a tail flick in a WT larva demonstrates the recruitment of a very small brain region during the low frequency transients of the LFP. Observe the movement of the head profile when the red mark appears. Video S6: X, y, t reconstruction of a binarized representation of the recruitment of brain territories during activity transients. Video S7: X, y, t reconstruction of the high Ca^2+^ clusters during activity transients. Video S8: X, y, t reconstruction of the high Ca^2+^ clusters during activity transients after low-pass filtering. The threshold for cluster display has been set to 300 voxels.
