# Supplementary material for: Evolution of Epileptiform Activity in Zebrafish by Statistical-Based Integration of Electrophysiology and 2-Photon Ca2+ Imaging
Source: Cells. 2020 Mar 21;9(3):769. doi: 10.3390/cells9030769 (PMC7140665; doi:10.3390/cells9030769)
Supplement: Supplementary file 1 [file cells-09-00769-s001.zip › supplementary figures/Figure S1.pdf]

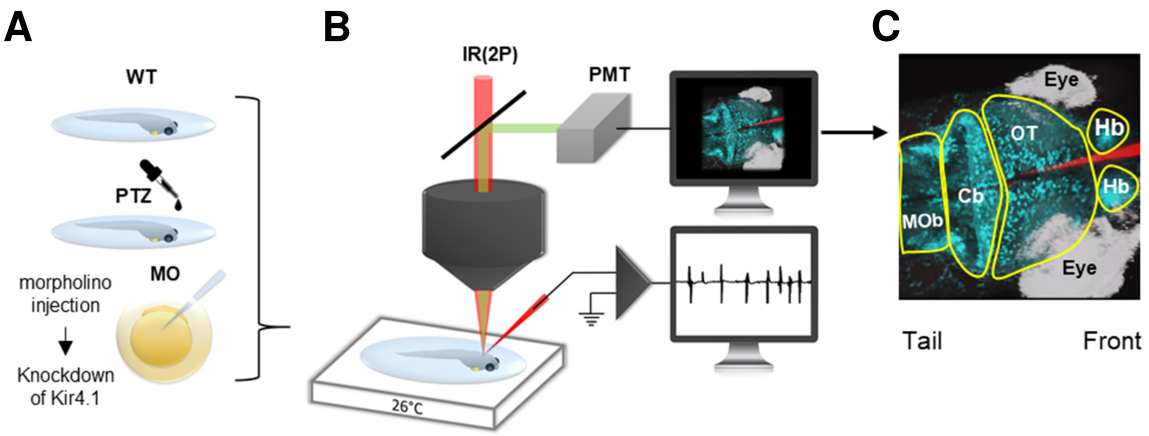

Fig. S1 Experimental model. (**A**) Five days old zebrafish larvae expressing the Ca^{2+} sensor GCaAMP6f were embedded in low melting point agar before LFP recordings and 2-photon imaging. Larvae developed pathological activity either because of acute treatment with PTZ or because of the reduced expression of the gene *kcnj10a* coding for a K^+ inward rectifier channel. (**B**) Larvae were transferred under the 2-photon microscope and the LFP was recorded with a glass microelectrode during Ca^{2+} imaging. (**C**) The imaged plane includes most of the zebrafish brain, including the Habenula (Hb), the Optic Tectum (OT), the Cerebellum (Cb) and the Medulla Oblungata in the back (MOb). The image field is 0.6 mm wide.
