# Supplementary material for: Evolution of Epileptiform Activity in Zebrafish by Statistical-Based Integration of Electrophysiology and 2-Photon Ca2+ Imaging
Source: Cells. 2020 Mar 21;9(3):769. doi: 10.3390/cells9030769 (PMC7140665; doi:10.3390/cells9030769)
Supplement: Supplementary file 1 [file cells-09-00769-s001.zip › supplementary figures/Figure S2.pdf]

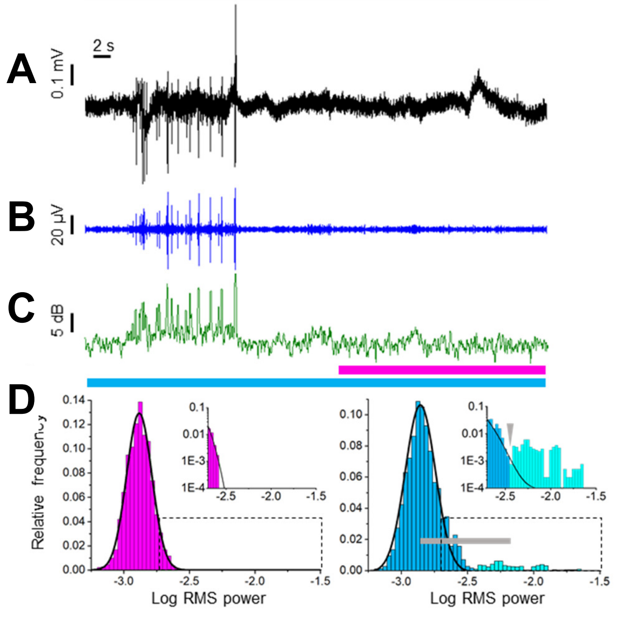

Fig. S2 Statistical analysis of the LFP signal. (**A**) Full band (0.1-1 kHz) LFP recording that shows an activity burst followed by regular background baseline activity. (**B**) Band passed data in the 30-95 Hz band. (**C**) Logarithm of the RMS power computed in a sliding window of 250 ms width. The burst is characterized by a clear power increase. (**D**) Distribution of spectral power during the resting baseline period (left panel) corresponding to the trace underlined by the magenta bar and during the entire acquisition period (right panel) corresponding to the trace underlined by the light blue bar. The baseline is characterized by a Gaussian distribution (black continuous lines) and the occurrence of the high frequency burst caused the emergence of a high energy tail on the right of the main mode (light blue samples in the right inset, the grey arrowhead indicates the 2 standard deviation limit). The power distribution returns three metrics: the average of the main mode, the difference between the average of the high energy tail and of the main mode (gray bar) and the relative frequency of the high energy events (cyan bars).
