# Supplementary material for: Evolution of Epileptiform Activity in Zebrafish by Statistical-Based Integration of Electrophysiology and 2-Photon Ca2+ Imaging
Source: Cells. 2020 Mar 21;9(3):769. doi: 10.3390/cells9030769 (PMC7140665; doi:10.3390/cells9030769)
Supplement: Supplementary file 1 [file cells-09-00769-s001.zip › supplementary figures/Figure S3.pdf]

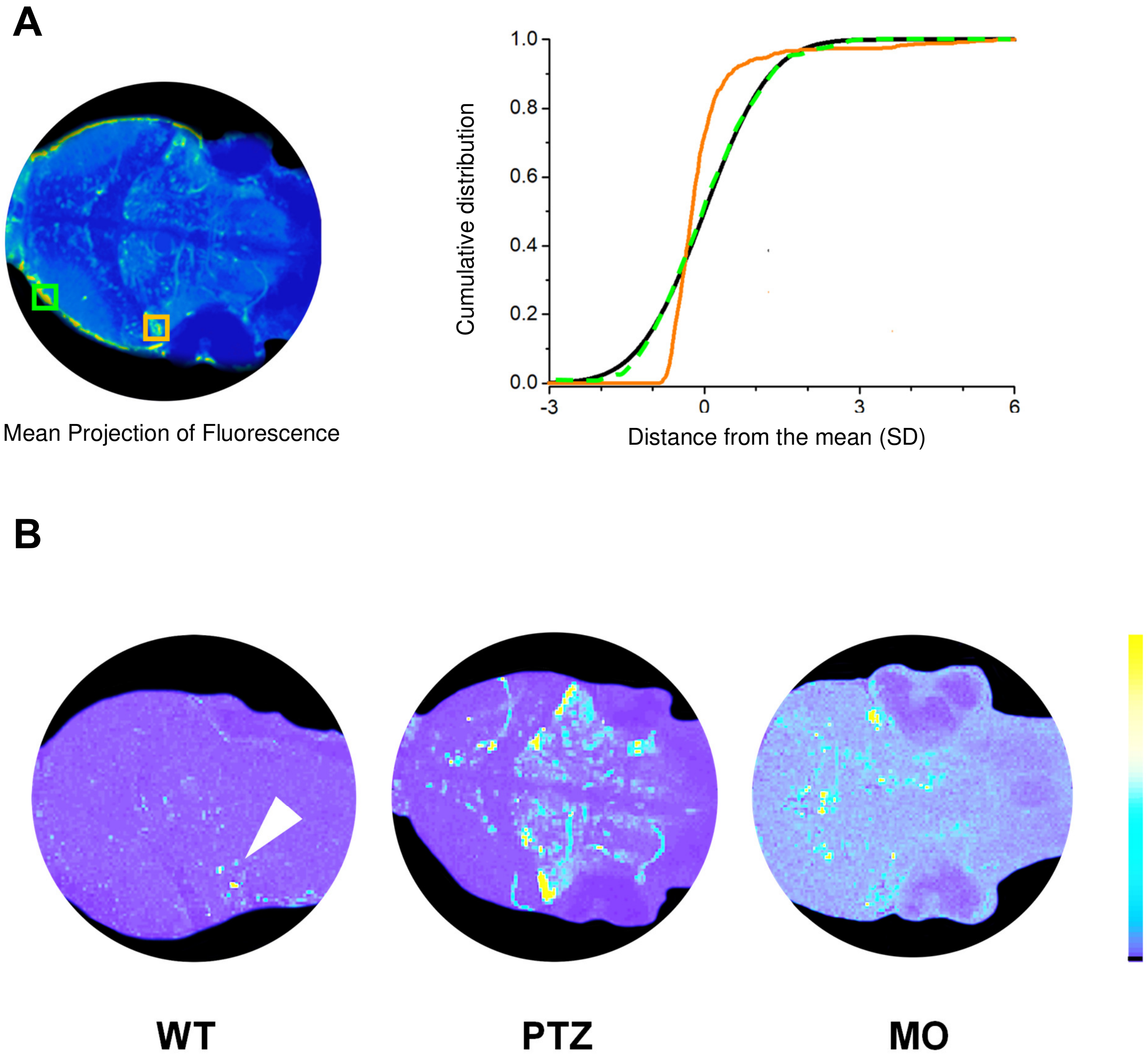

Fig. S3 Computation of the maps of Ca^{2+} activity. (**A**) Cumulative distribution of fluorescence fluctuations of two pixels compared to Gaussian cumulative distribution (black curve). The green pixel is representative of areas with fluorescence fluctuations depending only on noise (*i.e.* border of the fish) and its distribution is normal (dashed green). The orange pixel in the cerebellum has a skewed distribution because of the positive Ca^{2+} transients (orange). The more active is a pixel, the larger is difference between its cumulative distribution and the Gaussian cumulative distribution. (**B**) Example of a map for each model, where each pixel is color coded depending on the difference between its cumulative distribution and the Gaussian cumulative distribution. Cumulative distributions have been normalized to mean 0 and standard deviation 1.
