# Supplementary material for: Evolution of Epileptiform Activity in Zebrafish by Statistical-Based Integration of Electrophysiology and 2-Photon Ca2+ Imaging
Source: Cells. 2020 Mar 21;9(3):769. doi: 10.3390/cells9030769 (PMC7140665; doi:10.3390/cells9030769)
Supplement: Supplementary file 1 [file cells-09-00769-s001.zip › supplementary figures/Figure S4.pdf]

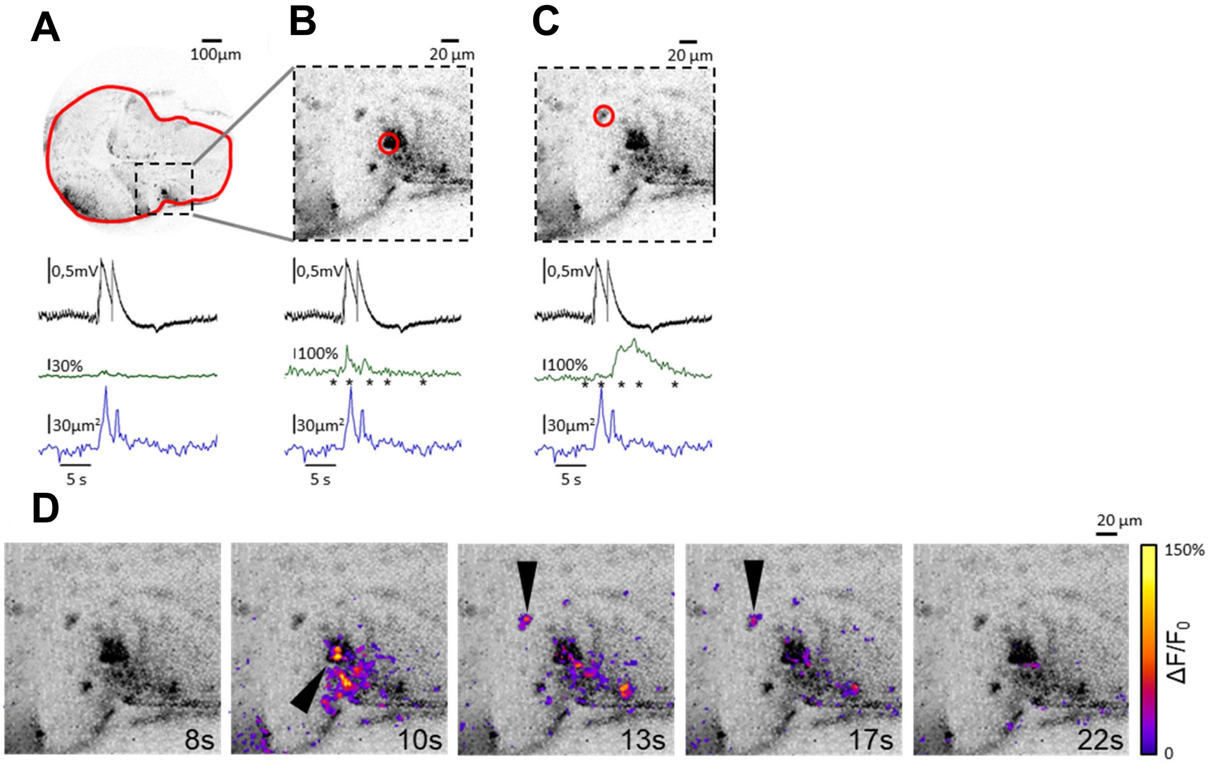

Fig. S4 Activity and motion. (**A, B, C**) Alignment of LFP (black), Ca^{2+} (green) and motion (blue) traces. The Ca^{2+} fluctuations have been averaged on the red regions displayed in the three panels. In panel **A**, the Ca^{2+} fluctuations are measured on the whole brain section. In panels B and C the Ca^{2+} fluctuations are measured on two different single neurons (red circles) that show motion-correlated activity. (**D**) $\Delta F/F_0$ images showing specific temporal points (*) of calcium traces in B and C.
