# Supplementary material for: Evolution of Epileptiform Activity in Zebrafish by Statistical-Based Integration of Electrophysiology and 2-Photon Ca2+ Imaging
Source: Cells. 2020 Mar 21;9(3):769. doi: 10.3390/cells9030769 (PMC7140665; doi:10.3390/cells9030769)
Supplement: Supplementary file 1 [file cells-09-00769-s001.zip › supplementary figures/Figure S5.pdf]

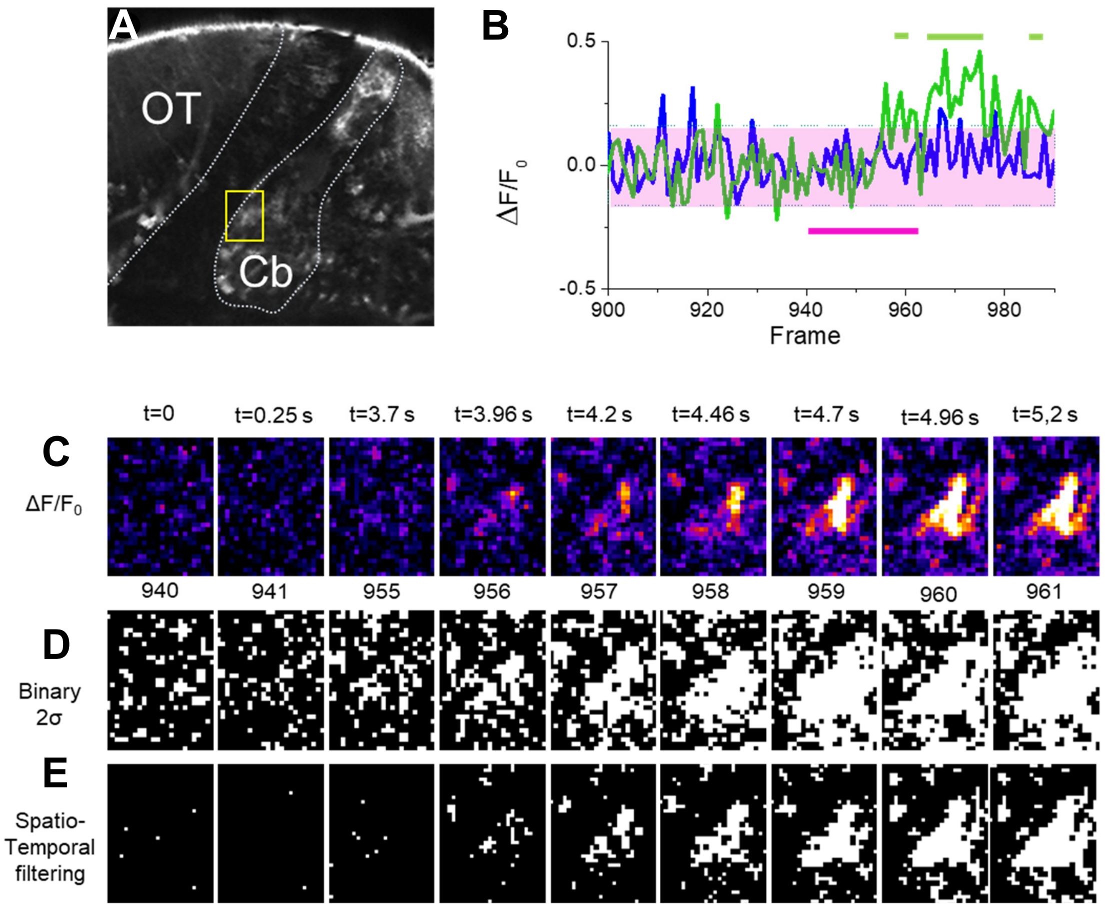

Fig. S5 Computation of the binarized imaging data. (A) The imaging data originates from a PTZ treated larva. The area shown here includes part of the right optic tectum (OT) and cerebellum (Cb). The yellow rectangle indicates the area magnified in the panels below (25 px wide). **(B)** Temporal profile of the $\Delta F/F_0$ of one pixel belonging to the Cb (green line) and one pixel outside the Cb area, 5 µm more rostral (blue line). Both processes have median 0 (see Material and Methods) and the standard deviation of the $\Delta F/F_0$ measured on the entire brain profile is 0.08. The pink shaded area indicates the 2σ interval around the median. **(C)** False color representation of the $\Delta F/F_0$ process in the area indicated in a. The period represented here covers about 5.4 s in correspondence of the magenta bar in panel B. **(D)** The $\Delta F/F_0$ process of each pixel is binarized by setting to 0 all points where the fluctuation is within the pink shaded area or negative and to 1 the remaining. Panel B shows that both processes satisfy the threshold conditions at several times. **(E)** The binarized stack is filtered by a 3D filter that removes isolated pixels. In this example the filtering condition requires that 1) at each frame a pixel is set to 1 if it is surrounded by at least 4 over-threshold neighbors in the same frame and 2) a pixel must remain over threshold for at least 3 consecutive frames. The green bars in panel B indicates frame that satisfy the latter condition for the green process, while no frame satisfy the minimal duration for the blue process.
