# Supplementary material for: Evolution of Epileptiform Activity in Zebrafish by Statistical-Based Integration of Electrophysiology and 2-Photon Ca2+ Imaging
Source: Cells. 2020 Mar 21;9(3):769. doi: 10.3390/cells9030769 (PMC7140665; doi:10.3390/cells9030769)
Supplement: Supplementary file 1 [file cells-09-00769-s001.zip › supplementary figures/Figure S6.pdf]

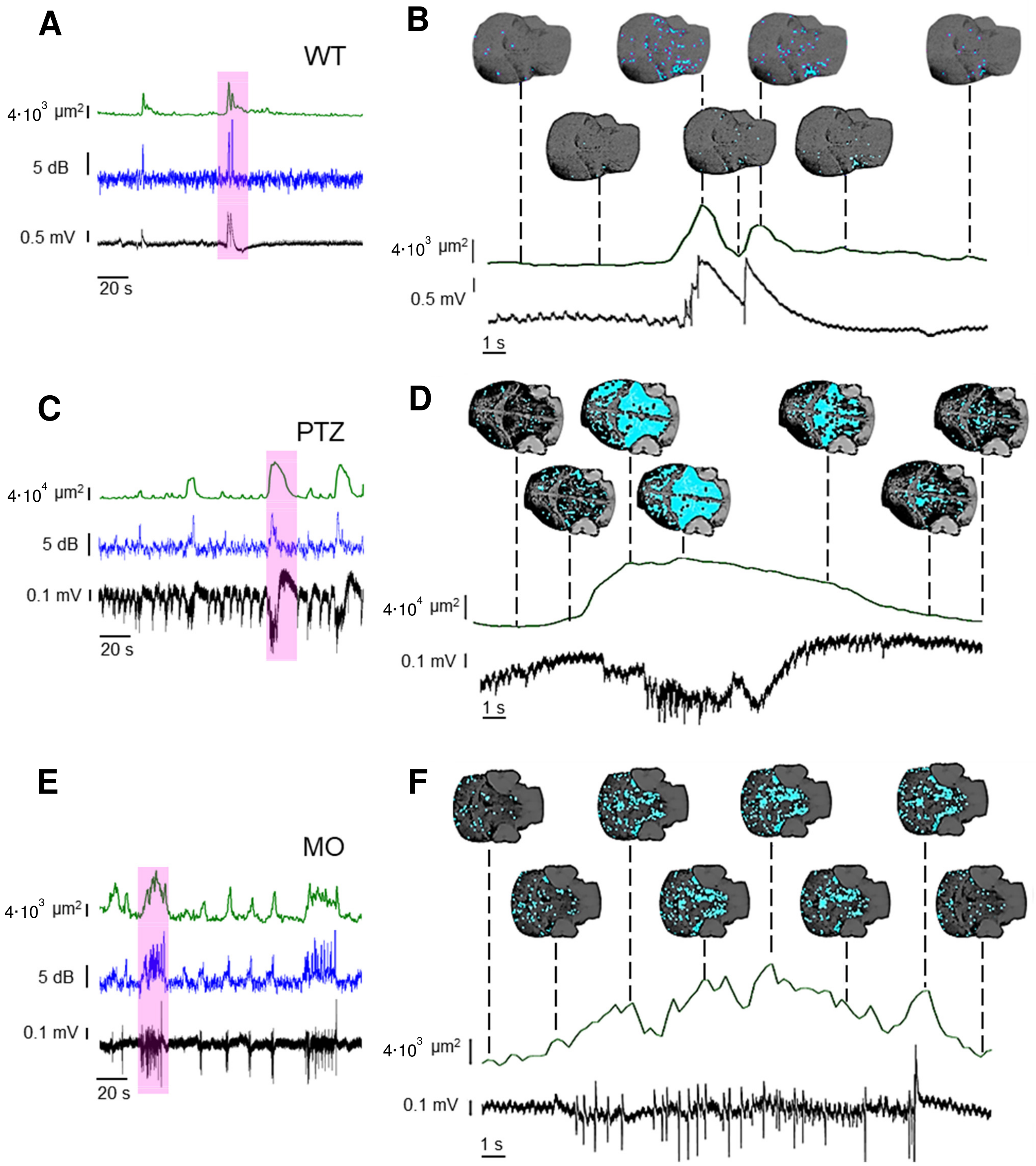

Fig. S6 Spatio-temporal evolution of the recruitment of high Ca^{2+} domains in the three experimental groups. (**A**) Representative recordings from control zebrafish. The green trace represents the temporal evolution of activity recruitment expressed as the surface of the high Ca^{2+} domains measured on the whole brain area. The blue trace reports the RMS power measured in the 30-95 Hz band. The black trace reports the raw LFP recording. Panels C and E show representative recordings for the PTZ treated larva (**C**) and for the *kcn10ja* morphants (**E**). (**B, D, F**) Magnification of the events highlighted in magenta in the traces on the left. Selected frames represent the recruited area (light blue pixels) aligned with the underlying traces.
