# Supplementary material for: Evolution of Epileptiform Activity in Zebrafish by Statistical-Based Integration of Electrophysiology and 2-Photon Ca2+ Imaging
Source: Cells. 2020 Mar 21;9(3):769. doi: 10.3390/cells9030769 (PMC7140665; doi:10.3390/cells9030769)
Supplement: Supplementary file 1 [file cells-09-00769-s001.zip › supplementary figures/Figure S7.pdf]

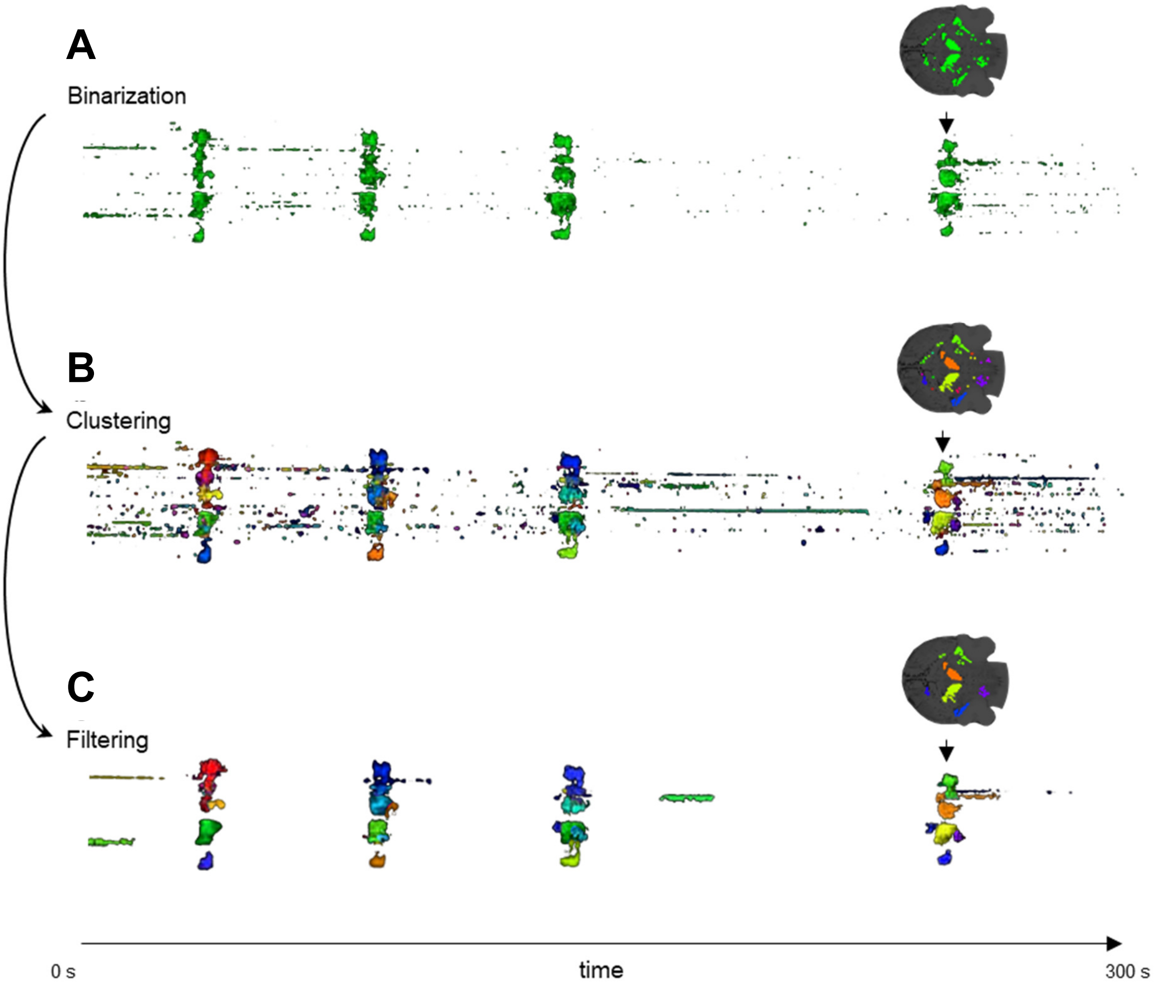

Fig. S7 Volume identification. **(A)** X,Y,T representation of the binarized Ca^{2+} fluctuations. **(B)** The binarized stack is clustered according to the proximity of voxels and each separate cluster is labelled in different colors. Each cluster is characterized by measured set of metrics: x,y,t volume, the surface of the x,y projection, the temporal duration. **(C)** By setting different dimensional thresholds it is possible to analyze just a sub-cluster of volumes. Here, are represented anly the clusters larger than 300 voxels
