# Supplementary material for: Evolution of Epileptiform Activity in Zebrafish by Statistical-Based Integration of Electrophysiology and 2-Photon Ca2+ Imaging
Source: Cells. 2020 Mar 21;9(3):769. doi: 10.3390/cells9030769 (PMC7140665; doi:10.3390/cells9030769)
Supplement: Supplementary file 1 [file cells-09-00769-s001.zip › supplementary figures/Figure S8.pdf]

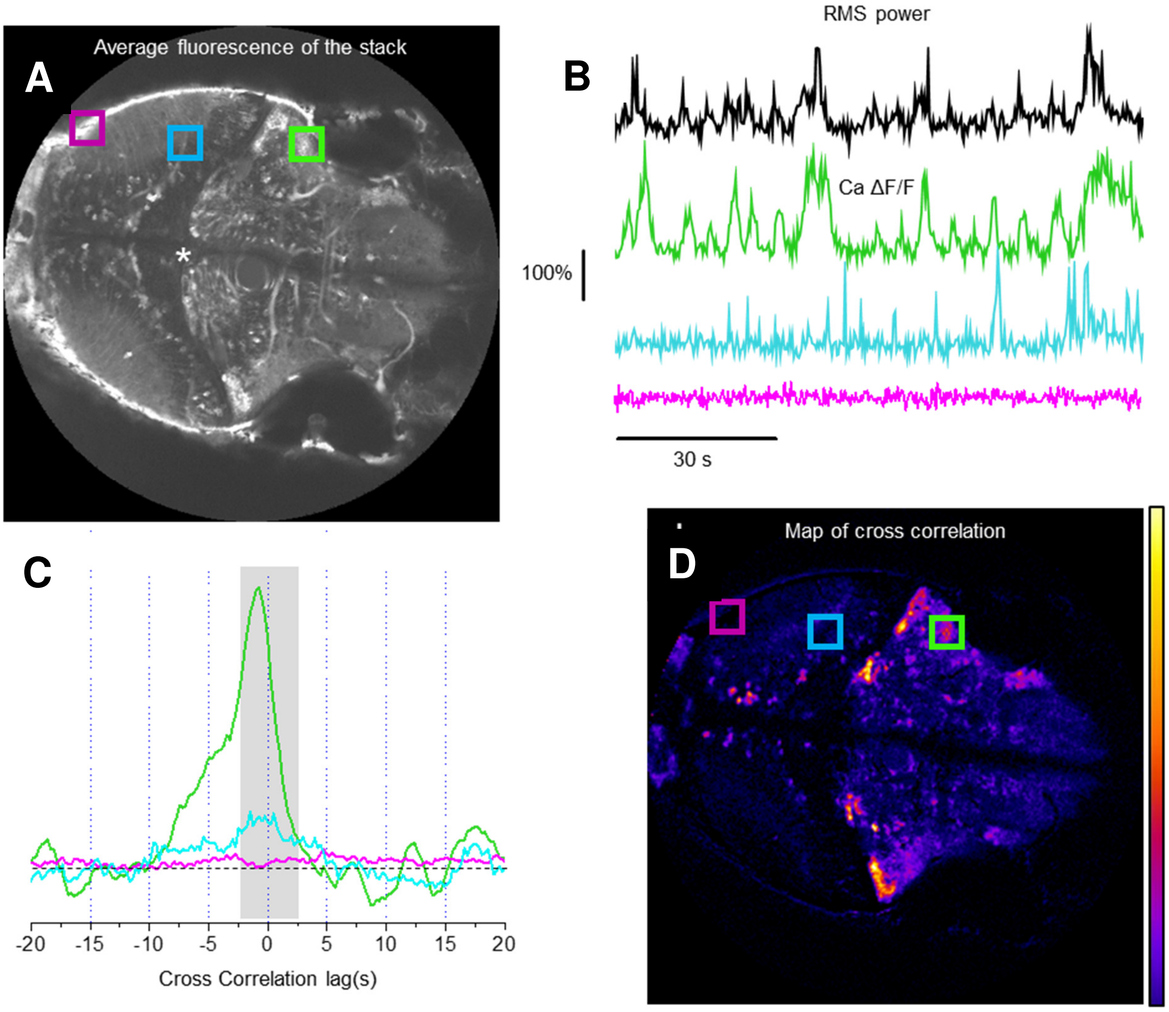

Fig. S8 Computation of the cross correlation maps. (A) Image obtained by averaging a time lapse stack of 1200 frames. The fluorescence fluctuations (have been measured in 3 areas of 2x2 pixels placed at the center of the three colored squares. The tip of the electrode recording the LFP is placed about 100 µm below the focal plane in the approximate location indicated by the white asterisk. **(B)** The black trace shows the temporal evolution of the RMS power of the LFP aligned with the calcium fluctuations recorded in the areas of the corresponding colours. The green area is centred on a portion of the cerebellum, the light blue area is in the optic tectum and it shows large transient fluctuations that are poorly correlated with the LFP activity transients. Finally, the magenta line indicates the fluctuations recorded at the edges of the skull in a region of high auto fluorescence. **(C)** Central part of the cross correlation spectra between the RMS power and the Ca^{2+} fluctuations recorded in the three areas: the area of the central peak is an accurate metric of the degree of temporal correlation between the LFP power and the activity of each area. The grey band indicates the area in which is computed the integral of the cross correlation spectra. **(D)** Cross correlation map of this time lapse sequence.
