# Supplementary material for: Evolution of Epileptiform Activity in Zebrafish by Statistical-Based Integration of Electrophysiology and 2-Photon Ca2+ Imaging
Source: Cells. 2020 Mar 21;9(3):769. doi: 10.3390/cells9030769 (PMC7140665; doi:10.3390/cells9030769)
Supplement: Supplementary file 1 [file cells-09-00769-s001.zip › supplementary figures/Figure S9.pdf]

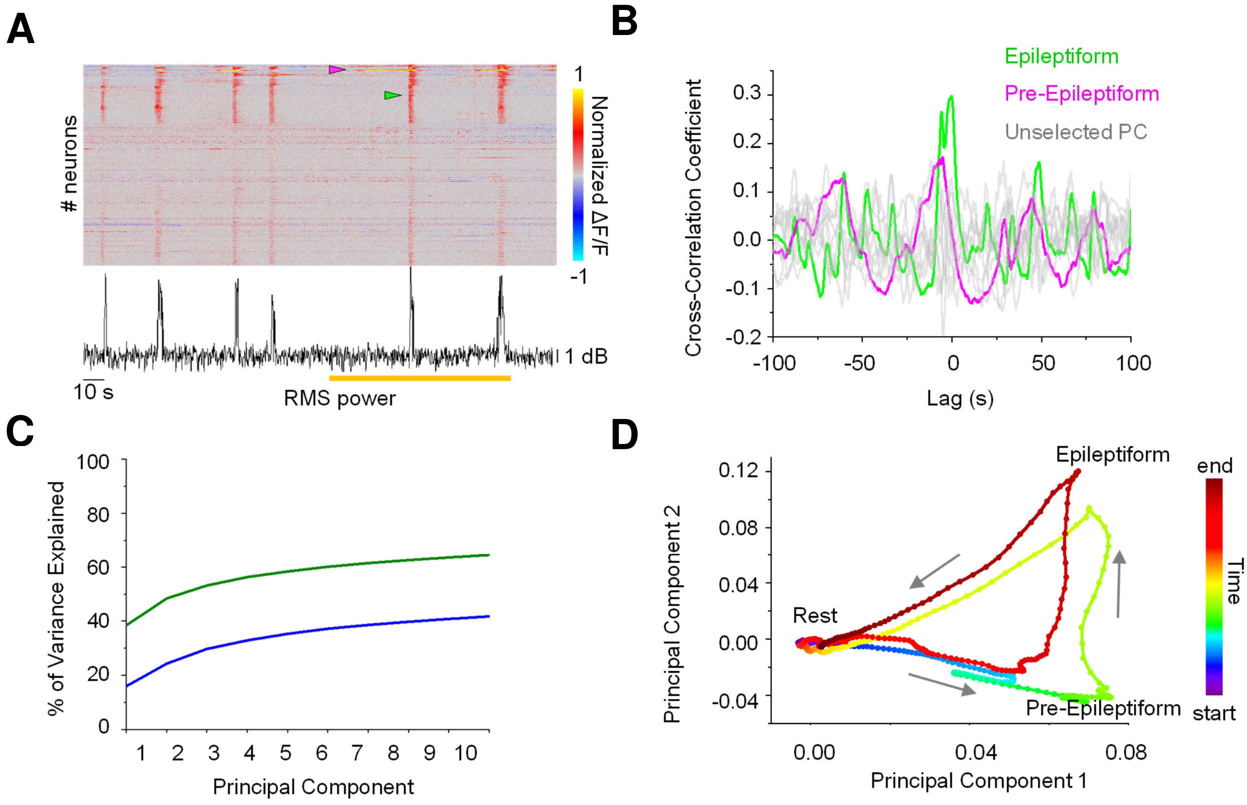

Fig. S9 PCA analysis (A) Raster plot of the normalized ΔF/F calcium activity for all neurons (n=350) identified in a time lapse sequence in the PTZ model. The magenta arrow indicates a neuron that increases its activity just before the epileptiform burst and the green arrow indicates a neuron that is active during the burst as represented by the RMS power in the 30-95Hz band (bottom black trace). **(B)** Cross-correlogram between the RMS power and the first 10 principal component. The one that present the highest value at lag zero is coloured in green while the one that present a positive peak just before the lag zero is coloured in magenta; the remaining in grey. **(C)** Percentage of the variance explained by each principal component sorted in a descending order. **(D)** Coordinated activity of the two selected components (shown in Fig. 6A) in the time period indicated by the yellow line in **A**. In the x-axis is represented the activity of the pre-Ep component while in the y-axes the Ep. The color-coding indicates the passage of time.
